# Progress toward malaria elimination in Jazan Province, Kingdom of Saudi Arabia: 2000–2014

**DOI:** 10.1186/s12936-015-0858-1

**Published:** 2015-11-09

**Authors:** Ibrahim M. El Hassan, Ahmed Sahly, Mohammed H. Alzahrani, Raafat F. Alhakeem, Mohammed Alhelal, Abdollah Alhogail, Adil A. H. Alsheikh, Abdullah M. Assiri, Tageddin B. ElGamri, Ibrahim A. Faragalla, Mohammed Al-Atas, Mohammed A. Akeel, Ibrahim Bani, Hussein M. Ageely, Abdulaziz A. BinSaeed, David Kyalo, Abdisalan M. Noor, Robert W. Snow

**Affiliations:** Faculty of Medicine, University of Jazan, Jazan, Kingdom of Saudi Arabia; Ministry of Health, Riyadh, Kingdom of Saudi Arabia; Public Health Directorate, Ministry of Health, Riyadh, Kingdom of Saudi Arabia; Spatial Health Metrics Group, Kenya Medical Research Institute-Wellcome Trust Research Programme, Nairobi, Kenya; Centre for Tropical Medicine and Global Health, Nuffield Department of Clinical Medicine, University of Oxford, Oxford, UK

## Abstract

**Background:**

The draft Global Technical Strategy for malaria aims to eliminate malaria from at least 10 countries by 2020. Yemen and Saudi Arabia remain the last two countries on the Arabian Peninsula yet to achieve elimination. Over the last 50 years, systematic efforts to control malaria in the Kingdom of Saudi Arabia has successfully reduced malaria cases to a point where malaria is now constrained largely to Jazan Province, the most south-western area along the Red Sea. The progress toward elimination in this province is reviewed between 2000 and 2014.

**Methods:**

Data were obtained from the Ministry of Health case-reporting systems, activity reports, unpublished consultants reports, and relevant scientific published papers. Sub-provincial population data were obtained the national household censuses undertaken in 2004 and 2010. Rainfall data were obtained from the Meteorological Department in Jazan.

**Results:**

Between 2000 and 2014 there were 5522 locally acquired cases of malaria and 9936 cases of imported malaria. A significant reduction in locally acquired malaria cases was observed from 2000 to 2014, resulting in an average annual incidence (2010–2014) of 0.3 cases per 10,000 population. Conversely imported cases, since 2000, remain consistent and higher than locally acquired cases, averaging between 250 and 830 cases per year. The incidence of locally acquired cases is heterogeneous across the Province, with only a few health districts contributing the majority of the cases. The overall decline in malaria case incidence can be attributed to coincidental expansion of control efforts and periods of exceptionally low rainfall.

**Conclusions:**

Jazan province is poised to achieve malaria elimination. There is a need to change from a policy of passive case detection to reactively and proactively detecting infectious reservoirs that require new approaches to surveillance. These should be combined with advanced epidemiological tools to improve the definitions of epidemiological receptive and hotspot malaria risk mapping. The single largest threat currently remains the risks posed by imported infections from Yemen.

## Background

The draft Global Technical Strategy for malaria 2016–2030 aims to eliminate malaria from at least 10 countries by 2020 [1]. Yemen and Saudi Arabia remain the last two countries on the Arabian Peninsula to achieve elimination [2–4]. Over the last 50 years, systematic efforts to control malaria in the Kingdom of Saudi Arabia has successfully shrunk the extent of *Plasmodium falciparum* and *Plasmodium vivax* risks. Starting with the oil rich areas in the Eastern region, the use of annual indoor residual house spraying (IRS) with dichlorodiphenyl-trichloroethane (DDT) and dieldrin, between 1948 and 1957, led to a dramatic decline in malaria case incidence [5] and local transmission was interrupted by 1975 [6]. From 1956, significant progress was made in reducing the malaria risks maintained by *Anopheles superpictus* through the application of DDT IRS and larvicides in the northern borders with Jordan and Iraq. Active transmission of malaria in the northern regions was interrupted in the 1970s [4, 6].

The hardest areas to control were located along the Red Sea, where *Anopheles sergentii* and *Anopheles arabiensis* sustained transmission [6, 7]. The pilgrimage routes used by those on the Hajj were protected through Abate^®^ larviciding and DDT IRS in rural households through the 1970s. Small residual foci remained in the lower reaches of the Hijaz mountains and persistent *An. arabiensis* foci in the foothills of Mecca [6, 8]. Malaria control activities in the south-western regions of the Kingdom did not start until 1972.

Epidemics were reported during the mid-late 1990s in the south-western regions of the Kingdom and malaria case incidence began to rise [4, 9, 10]. By 2003, more than 70 % of the total national case burden occurred in this region, mainly Jazan and Asir Provinces. Renewed efforts to achieve malaria pre-elimination were launched in 2004. The last 10 years have witnessed significant declines in malaria in the south-western provinces. Malaria transmission was eventually constrained to Qunfudha and Al Lith sectors of Mecca Province, Asir and Jazan Province. In 2009, only 61 autochthonous cases were reported and all came from foci in Jazan and Asir [11].

The objectives of the National Malaria Strategy 2004–2007 were laid out to serve as a pathway to pre-elimination with a concentrated effort in the Southern region and enhanced surveillance in other previously controlled areas to sustain a malaria free state. This paper reviews the case data at a granular level and the elimination activities mounted between 2000 and 2014 in Jazan Province.

## Methods

### Location and ecology

Jazan Province is one of the smallest (11,670 km^2^) administrative areas of the Kingdom, located in the south-western tip of the country with a coastal boundary 260 km along the Red Sea and a 120 km border with the Republic of Yemen. The Yemeni border was finally officially ratified following disputed 1934 boundaries in June 2000 as part of the Jeddah Treaty [12]. The population, according to the 2010 census, was 1.37 million.

**Fig. 1:**
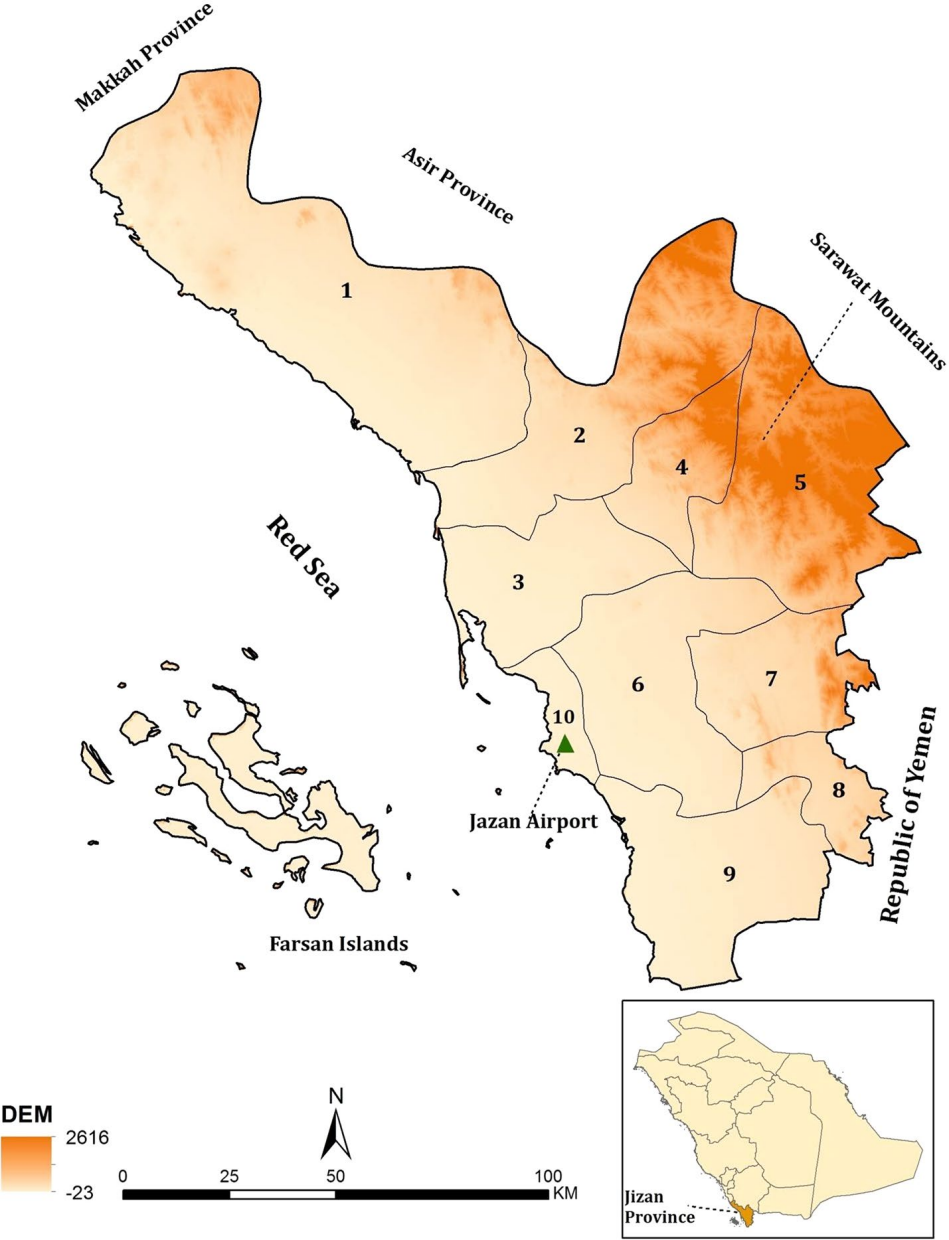
The topography and boundaries covered by malaria centres in Jazan Province. *1* Darb/AdDarb/Aldarab, *2* Baish/Bayesh/Bayish and Elrith, *3* Sabya, *4* Harub, *5* AlEdabi/Eleidaby, *6* Abu Arish, *7* Al Aridah/Alaadrda, *8* Al khoba, *9* Samtah, *10* Jazan/Farasan Islands. Digital elevation data in metres above sea level

The province includes over 100 islands located in the Red Sea, including the Farasan Islands. The Al-Sarawat mountains rise to over 3000 m, the highland areas of the Fayfa Mountains lead to plains (Fig. 1) that provide rich escarpment agriculture, including sorgo, coffee, millets, apples, mangoes and citrus fruits [13]. The coastal regions are part of the Tihama straights that extend down through Yemen and are hot and arid.

The climatic and environmental conditions of the region are suitable for transmission of tropical diseases, including soil-transmitted helminths, schistosomiasis and insect-borne infectious diseases [14, 15]. Currently the Jazan region is witnessing significant development in all sectors, especially the health and educational sectors, in accordance with the marked growth in the economy.

### Historical malaria potential and control

During the Precambrian age, Jazan Province was part of the African continent and the vegetation and insect ecology of this region is closely related to that of East Africa and lying within the Afro-tropical zone. Two principal malaria vectors exits in Jazan, *An. sergentii*, located more frequently on border with Asir Province in the north, and *An. arabiensis* more commonly associated with transmission closer to Yemeni border. Other Anopheline species identified in the province but never implicated in malaria transmission include *Anopheles d’thali*, *Anopheles rupicolus*, *Anopheles multicolor*, *Anopheles turkhudi*, *Anopheles pretoriensis* and *Anopheles tenebrosus* [16–21].

The *Plasmodium falciparum* prevalence rate among 370 school children attending 13 schools in Jazan province in 1976 was 4.9 % [22]; in 1984 prevalence was 1.4 % among 6384 school children from 30 schools [23]. The only other parasite detected was *Plasmodium malariae*, one in 1976 and one in 1984. Thirty years ago the area was probably best described as hypoendemic.

Although there are some reports of larviciding in the town of Jazan in 1973, vector control did not start until the early 1980s [24]. From 1983, the malaria control programme in Jazan focussed on attacking the adult vectors using annual indoor residual spray (IRS) rounds of DDT in pre-defined areas of endemic risk accompanied by Abate^®^ (Temphos 50EC) larviciding in persistent foci covering *circa* 800 villages [25]. After 1 year of aggressive vector control, and reductions in slide positivity, the regional control programme abandoned IRS in favour of only larviciding. Ultra-low volume (ULV) spaying was undertaken using the pyrethroid Reslin^®^ in some selected persistent foci in the mid-1980s [25]. Investment in malaria control financing and staff were reduced during the late 1980s. Epidemics were recorded between 1986–1988, 1992, 1995–1996 and the severe epidemic of 1997–1998, lead to renewed efforts at vector control accompanied by large increases in funding made available to the Jazan malaria programme. DDT resistance was detected in 1987, resulting in a malaria outbreak in 1988. DDT was replaced the same year with the organophosphates fenitrothion and malathion for IRS with two annual rounds aimed at blanket spraying. In 1995, the preferred insecticide for IRS was switched to the pyrethroid lambdacyhalothrin 10 CS.

### Developing intervention context since 2000

Ministry of health reports, records, unpublished consultants reports and published scientific papers were reviewed to create a timeline of major policy changes for malaria case treatment, adult and larval vector control and case-detection methods. Where possible information on numbers of houses sprayed, populations protected by IRS, numbers of insecticide-treated nets (ITN) distributed, areas (km^2^) covered by larvicidings and numbers of foci of locally acquired infections investigated were recorded for each year since 2000.

The susceptibility of vectors to applied insecticides was periodically measured before 2000, but more routinely since 2005 and the results of 24 h delayed mortality from standard bioassays for the major insecticide classes were recorded from Ministry of Health reports. Longitudinal data related to anti-malarial drug sensitivity before 2004, when the Ministry of Health, implemented artemisinin-based combination therapy, is scanty but information has been assembled on treatment failures and molecular markers of resistance from the published literature. Finally, information related to major political milestones, notably border conflicts and refugees were assembled separately from a variety of sources and local knowledge.

### Assembling malaria case data

The National Malaria Control Service established a national malaria training centre in Jazan in 1981, supporting eight malaria reconnaissance centers along the Thiama straits from 1983. This was extended in 2001 to cover nine reporting areas (Fig. 1). Jazan and the Islands of Farasan are not covered by special malaria centres.

For the last 20 years passive case detection (PCD) has been maintained through a very comprehensive system of primary health centres (174) and hospitals (21) across the Province. Parasitological diagnosis of malaria using microscopy is provided free-of-charge. Rapid diagnostic tests (RDTs) were introduced by the Malaria Department in 2004. The data from the health system form the basis of case-incidence reporting, but are supplemented by reactive active case detection (ACD) data from detected foci in the nine malaria centres. Proactive ACD though mass blood surveys (MBS) started early in 1982 for epidemiological stratification, these were suspended for several years until annual MBS were resumed in 2004. Data are reported to the malaria department headquarters at the Jazan Health Affairs Department, which are forwarded to the National Malaria Authority at Ministry of Health in Riyadh. In Jazan, new epidemiological surveillance forms were developed in 2008 to include the compulsory blood examination of arriving foreign labourers. Here all confirmed cases reported by each of the nine malaria reference areas and PCD from Jazan and Farasan areas from January 2000 to December 2014 were assembled.

### Assembling population and rainfall data

National household censuses were conducted in 2004 and 2010. In Jazan Province these covered 14 census, administrative areas. These administrative areas were reconciled to ten malaria reporting centres (nine established centres and Jazan/Farasan combined). The malaria reporting centres of Sabiah and Haroub spanned one census administrative area (Sabiah) and the populations in these two malaria reporting areas have been assumed to be equivalent (half of the reported total population in Sabiah). Annual inter-censal growth rates were computed for each of the ten areas and used to extrapolate population totals across all surveillance years 2000–2014. Monthly rainfall data were obtained from Jazan Airport (Fig. 1) covering the period January 2000 to December 2014.

## Results

### Changing malaria treatment practices

During the early 1980s, chloroquine (CQ) remained an effective treatment for both *P. falciparum* and the rarer cases of *P. vivax*. CQ resistant *P. falciparum* infections and treatment failures began to be documented in the Kingdom from 1992 [26, 27]. From 1998, CQ resistance escalated [26, 28] and by the mid-2000s the prevalence of molecular markers of CQ resistance were above 80 % [29–31]. As CQ began to fail there was increasing use of the second-line recommended drug, sulfadoxine-pyrimethamine (SP) for treatment. CQ was officially replaced with artesunate-SP (AS-SP) as first-line treatment and artemether-lumefantrine (AL) as a second-line treatment in 2007 [32]. By 2008, the double mutant associated with dihydrofolate reductase in *P. falciparum* infections conferring resistance to SP was found in over 70 % of infections in Jazan [31, 33]. In practice, both artemisinin-based drug combinations, AQ-AP and AL, were used before this date and interchangeably as first/second line treatments since this date. During the 1980s, CQ plus primaquine (PQ) was introduced as a gametocyticidal treatment for *P. falciparum* and radical cure for *P. vivax* infections. PQ use was suspended in 2004, when AQ-SP and AL were introduced, however its use as a radical treatment has been re-introduced in the last 2 years, without screening for G6PD deficiency.

### Vector control

Abate was used as a larvicide for many years in Jazan. Insecticide susceptibility tests conducted in 2002 showed reduced sensitivity to *Culex tritaeniorhynuchus* (one of vectors of Rift valley fever) and larviciding was switched to biological control using *Bacillus thuringiensis israelensis*, insect growth regulators [diflubenzuron (25 %) and pyriproxyfen (0.5 %)] and to a lesser extent chemical control using pyridafenthion. Larviciding is routinely undertaken in 103 wadis (valleys) and 534 tributaries carrying water all the year round with total length of 3469 km. Additional larviciding activities are undertaken at 13,800 breeding sites inside focal transmission villages.

Lambdacyhalothrin 10 CS was used for IRS between 1995 until 2003 when a switch was made to deltamethrin WG 250, within the pyrethroid insecticide class. Between 2011 and 2014, lambdacyhalothrin 10 CS was re-introduced. Since 2000, spraying occurs three times every 12 months in September, December and March. Communities identified for IRS are those where known or emerging foci have been described, covering approximately 1200 villages. Over the period 2004–2014, between 51,000 and 63,000 households have been protected by IRS, amounting to 83–93 % of target households and protecting *circa* 550,000 people. Bioassay data for deltamethrin and lambdacyhalothrin have shown consistently high 24-h mortality rates and have been fully susceptible between 2004 and 2014.

ITN have been available in the Kingdom for malaria control, provided free of charge, since the late 1980s [34]. Between 2000 and 2006 *circa* 500,000 ITN were distributed to communities in Jazan. Approximately 50,000 long-lasting insecticide-treated nets have been distributed, each year, between 2007 and 2012; these have been targeted to active foci of transmission and all communities along the border with Yemen.

### Declining case incidence

**Fig. 2:**
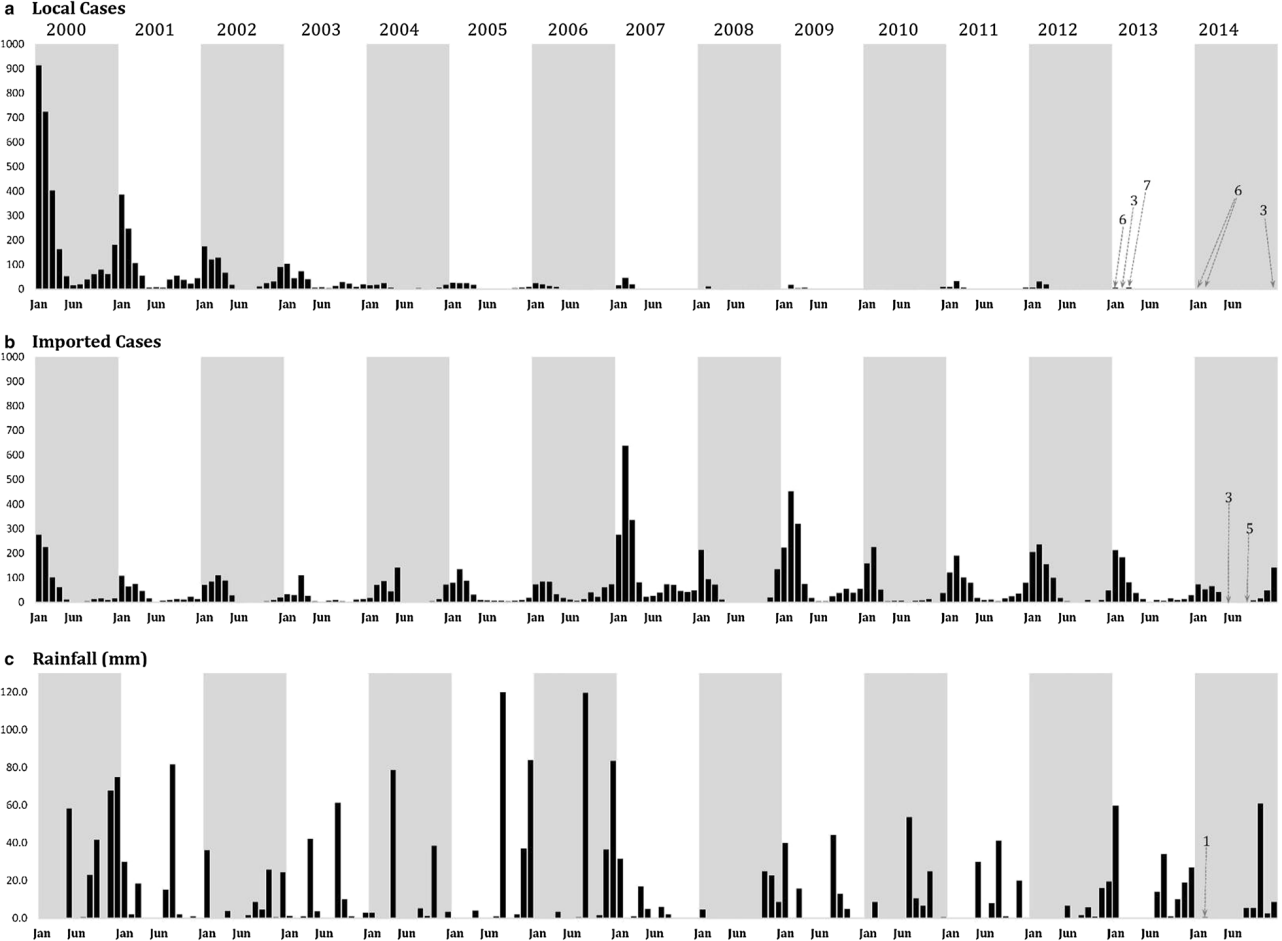
**a** Monthly, locally acquired malaria case burdens January 2000 to December 2014 reported through provincial passive case detection (PCD) systems and supplemented by reactive active case screening detection and since proactive ACD since 2004. **b** Monthly, imported malaria case burdens January 2000 to December 2014 reported through PCD systems and reactive ACD screening, proactive ACD since 2004 and screening of immigrants since 2008. **c** Monthly rainfall (mm) recorded at Jazan Airport (Fig. 1) from January 2000 to December 2014

Locally acquired cases declined significantly from 2000 (2756; 35.3 per 10,000 population) to 2004 (126; 1.4 per 10,000 population) (Fig. 2a). There continued to be a steady decline through to 2010 with only 18 locally acquired cases reported (0.15 per 10,000 population). In 2011, (58 cases) and 2012 (67 cases) a rise in local cases was observed (Fig. 2a), but declined again to very low numbers in 2013 (16 cases) and 2014 (15 cases), *circa* 0.11 per 10,000 population. The average annual incidence (2010–2014) of locally acquired cases reported through the PCD system and additional MBS was 0.3 per 10,000 population.

**Fig. 3:**
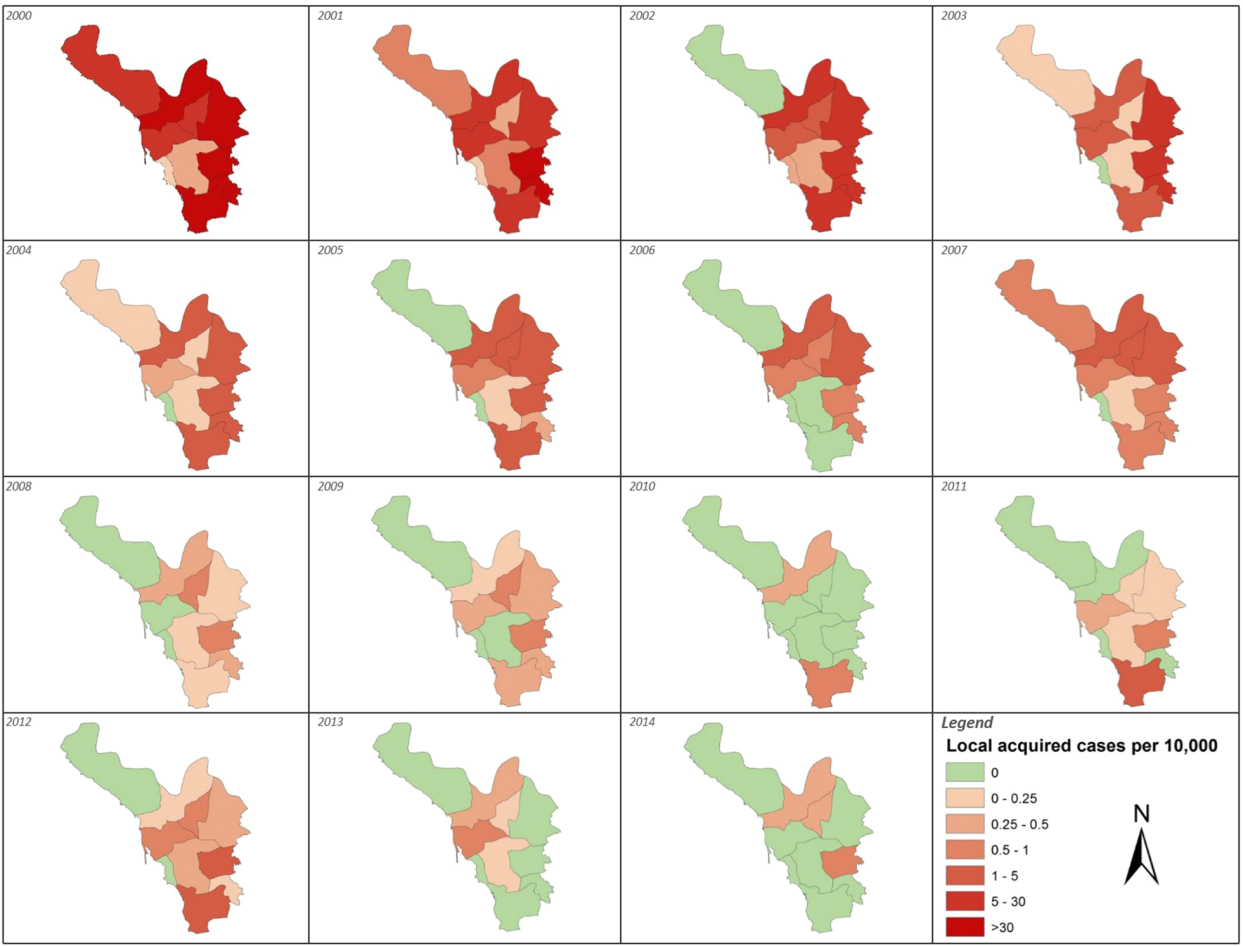
Annualized rates of locally acquired malaria case-incidence for each of the nine malaria reporting regions shown in Fig. 1 and Jazan/Farasan per 10,000 population for each year 2000–2014

The overall declines in autochthonous case incidence across the province masks year-to-year heterogeneities within the province. Rates of reported local case incidence for each of the 15 years of surveillance are shown in Fig. 3. The region of AdDarb, in the most northern reaches of the province, has experienced the least locally acquired malaria over the surveillance interval. Between 2001 and 2007 malaria incidence was highest in the regions bordering Yemen and Bayish/Elrith in the centre of the region. From 2008, when overall incidence had declined to very low levels the spatial distribution of locally acquired cases became more random between malaria reporting districts (Fig 1) with most years showing highest incidences in the Alaadrda, Samtah and Sabya areas of the region, sustained case incidence has been documented in Bayish/Elrith and Harub areas and Samtah has contributed 61 % of all locally acquired cases since 2010 (Fig. 3).

**Fig. 4:**
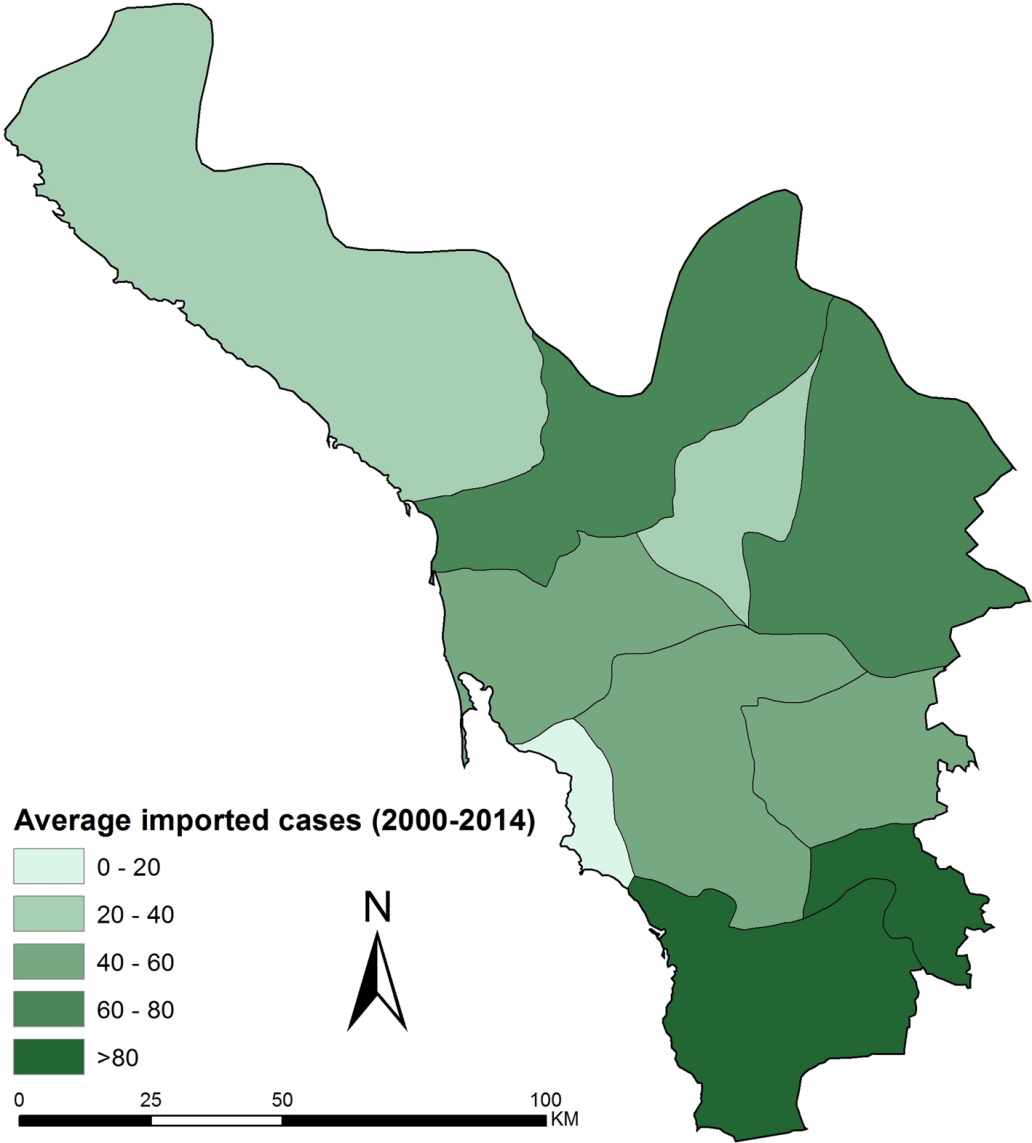
Average (2000–2014) annual imported malaria cases (*number*) recorded for each of the nine malaria reference regions and Jazan/Farasan

The largest case burden documented in the Province remains those defined as imported cases (Fig. 2b). Between 2000 and 2014 there were between 250–830 imported cases each year with two exceptional years: 2007 (1705 cases) and 2009 (1310 cases). Of note is that the seasonal patterns seen for locally acquired cases match those reported for imported cases. Most cases are imported from Yemen which shares a similar malaria seasonal ecology, and/or the seasonal importation of infections may explain the seasonal incidence of locally acquire cases due to local onward transmission from imported infections. The majority of imported cases have been described in Al Khoba and Samtah regions of the Province, bordering Yemen (Fig. 4).

### Rainfall and case-incidence

Rainfall is typically acutely seasonal and sporadic in Jazan (Fig. 2c). Two major precipitations in August of 2005 and 2007 did not, however, lead to significant epidemics as witnessed during the El Niño years of 1997/1998. It is notable that the period 2007–2014 was drier than the period 2000–2006, corresponding with lower case-incidence in the second interval.

## Discussion

In the south-western regions of the Kingdom of Saudi Arabia, the 1990 s were characterized by failing efficacies of drugs and insecticides, exceptional rainfall and declining attention, and financing for, malaria control. The consequence was that, despite earlier successes in reducing case-incidence in these regions, malaria began to rise in the 1990s and resulted in a series of epidemic outbreaks between 1992 and 1998, mainly in Jazan and Asir Provinces. During the early 2000s, malaria case incidence remained high in Jazan Province (Fig. 2a). Most notable was the high case-incidence in 2000, when for the first time outside of the African continent, there was a coincidental epidemic of the epizootic mosquito borne Rift Valley Fever in Yemen and Asir and Jazan Provinces of the Kingdom of Saudi Arabia [35].

From 2004, with Saudi Government commitment to eliminate malaria and improved strategic direction, funding and organization of the malaria programme, locally acquired malaria cases began to significantly decline (Fig. 2a). Mass blood surveys, using mainly microscopy in addition to RDTs in some areas, between 2012 and 2014 sampled 91,676 people across the province and found only three infections (0.003 %), all imported from Yemen (Tageddin ElGamri, personal communication), compared to MBS during the period 1978–1980, when the parasite rate was 6 % [25]. These observations imply that the reduction in case incidence has been accompanied by significant reductions in local parasite transmission.

It is not possible to make any direct attribution to specific interventions that will have contributed to the declining malaria across the Province; however there are several temporal observations that should be considered in understanding the malaria transition. First, declining case-incidence coincides with scaled insecticidal control targeted at larval and adult stages of Anopheles vectors and the increased distribution of ITN. This is an important association when compared to the counterfactual of reduced vector control in the 1980s and high case-incidence. Sustaining this impact, however, depends largely on continued financial support and militating against the ever-present threats of pyrethroid resistance. Both nets and house spraying depend entirely upon pyrethroid insecticides and while bioassay results suggest a continued high efficacy, switching insecticide classes will be important to reduce the risks of emerging resistance, or replace IRS with other classes of insecticides when resistance becomes established. Current approaches to IRS and larviciding are based upon blanket coverage of targeted areas of known high receptive risks. As case-incidence declines a more data informed basis of targeted control is required [36]. The case-incidence of malaria is not uniform across the province (Fig. 3), only five regions contribute 95 % of the locally acquired case burden in the province since 2010. A more stratified approach to vector control using reactive ACD and vector control based on localities of defined disease emergence in regions where case incidence is exceptionally low [37, 38]. This would reduce the insecticide pressure on local vector populations across the province.

Second, areas where the highest locally acquired case incidence are described are coincidental with those that also describe the highest numbers of imported cases (Figs. 3, 4). Importation of malaria from neighbouring Yemen continues to pose a significant threat to elimination in Jazan province. In 2001, the National Malaria Control Programme in Yemen was re-established and a collaborative programme to control cross-border malaria was resurrected and revised since it was first established in 1979. In 2007, an agreement to support efforts for a malaria-free Arabian Peninsula was signed by Ministers from both countries [39], a year when the largest number of imported cases was described (Fig. 2b). Political instability in Yemen led to refugees crossing into Jazan in 2009, resulting in a large number of reported imported malaria cases (Fig. 2b). Since 2012, with continued instability in Yemen the border with Jazan has been patrolled more rigorously, however the importation rate remains significant (Figs. 2b, 4).

Finally, the region has been subject to malaria, and other vector-borne disease, epidemics consequent upon rainfall anomalies. Rainfall in this area is subject to acute short-term temporal, seasonal fluctuations and long-term anomalies [40]. Since 2002, there has been a protracted drought, witnessed across the sub-region [41] and most notable in the period after 2007 in Jazan (Fig. 2c). Any future aberrations in rainfall pose threats to gains made in systematic vector control in this area, as exampled during the El Nino led epidemics in the late 1990s. One caveat to the analysis presented here is the use of only one meteorological station located in the city of Jazan. It is very likely that there are important variations in rainfall across the province and these warrant further investigation using higher resolution, temporal data from satellite imagery.

Present milestones of success in Jazan province are largely based on the numbers of disease events detected through PCD across its extensive free health service. The province remains in the pre-elimination phase and needs to migrate from detecting disease events to detecting infection events, draining the last residual foci of infection sinks and preventing the importation of new infections [36–38, 42]. This will require changes to how infections are identified. While rapid diagnostics and microscopy continue to be valuable tools for diagnosis, they lack sensitivity when the ambition is to identify all infections, including asymptomatic infections [43]. During a recent Mass Screening and Treatment pilot on the island of Zanzibar, RDTs detected 20 infections compared to 339 detected from the same population using polymerase chain reaction (PCR) [44]. Missed infections using microscopy have also been reported when compared to PCR analysis in Jazan [45]. Mass blood surveys should begin to use PCR techniques to detect sub-microscopic infections, these MBS should form part of either reactive case detection methods or proactive case detection methods based upon improved epidemiological receptive and hotspot risk mapping [37, 46–50].

The Saudi Government are fully committed to the malaria elimination end game committing US$ 30 million per year to this ambition, where more than 35 % of this budget is allocated for malaria control programme in Jazan. To achieve elimination will require a substantial increase in the use of epidemiological methods to predict, prevent, detect and contain new infections in the province.
